# Variants in *PRKAR1B* cause a neurodevelopmental disorder with autism spectrum disorder, apraxia, and insensitivity to pain

**DOI:** 10.1038/s41436-021-01152-7

**Published:** 2021-04-08

**Authors:** Felix Marbach, Georgi Stoyanov, Florian Erger, Constantine A. Stratakis, Nikolaos Settas, Edra London, Jill A. Rosenfeld, Erin Torti, Chad Haldeman-Englert, Evgenia Sklirou, Elena Kessler, Sophia Ceulemans, Stanley F. Nelson, Julian A. Martinez-Agosto, Christina G. S. Palmer, Rebecca H. Signer, Undiagnosed Diseases Network, Marisa V. Andrews, Dorothy K. Grange, Rebecca Willaert, Richard Person, Aida Telegrafi, Aaron Sievers, Magdalena Laugsch, Susanne Theiß, YuZhu Cheng, Olivier Lichtarge, Panagiotis Katsonis, Amber Stocco, Christian P. Schaaf

**Affiliations:** 1 Institute of Human Genetics, Heidelberg University, Heidelberg, Germany; 2 Faculty of Medicine, University of Cologne, Cologne, Germany; 3 Institute of Human Genetics, University Hospital Cologne, Cologne, Germany; 4 Section on Endocrinology and Genetics, Eunice Kennedy Shriver National Institute of Child Health and Human Development, Bethesda, MD, USA; 5 Department of Molecular and Human Genetics, Baylor College of Medicine, Houston, TX, USA; 6 Baylor Genetics Laboratory, Houston, TX, USA; 7 GeneDX, Gaithersburg, MD, USA; 8 Mission Fullerton Genetics Center, Asheville, NC, USA; 9 Department of Pediatrics, University of Pittsburgh School of Medicine, Pittsburgh, PA, USA; 10 Genetics/Dysmorphology, Rady Children’s Hospital, San Diego, CA, USA; 11 Department of Human Genetics, David Geffen School of Medicine at UCLA, Los Angeles, CA, USA; 12 Department of Psychiatry & Biobehavioral Sciences, David Geffen School of Medicine at UCLA, Los Angeles, CA, USA; 13 Institute for Society and Genetics, UCLA, Los Angeles, CA, USA; 14 Division of Genetics and Genomic Medicine, Department of Pediatrics, Washington University School of Medicine, Saint Louis, MO, USA; 15 Biosciences Institute, Faculty of Medical Sciences, Newcastle University, Biomedicine West Wing, International Centre for Life, Times Square, Newcastle upon Tyne, UK; 16 INTEGRIS Pediatric Neurology, Oklahoma City, OK, USA

## Abstract

**PURPOSE:**

We characterize the clinical and molecular phenotypes of six unrelated individuals with intellectual disability and autism spectrum disorder who carry heterozygous missense variants of the *PRKAR1B* gene, which encodes the R1β subunit of the cyclic AMP-dependent protein kinase A (PKA).

**METHODS:**

Variants of *PRKAR1B* were identified by single- or trio-exome analysis. We contacted the families and physicians of the six individuals to collect phenotypic information, performed in vitro analyses of the identified *PRKAR1B*-variants, and investigated *PRKAR1B* expression during embryonic development.

**RESULTS:**

Recent studies of large patient cohorts with neurodevelopmental disorders found significant enrichment of de novo missense variants in *PRKAR1B*. In our cohort, de novo origin of the *PRKAR1B* variants could be confirmed in five of six individuals, and four carried the same heterozygous de novo variant c.1003C>T (p.Arg335Trp; NM_001164760). Global developmental delay, autism spectrum disorder, and apraxia/dyspraxia have been reported in all six, and reduced pain sensitivity was found in three individuals carrying the c.1003C>T variant. *PRKAR1B* expression in the brain was demonstrated during human embryonal development. Additionally, in vitro analyses revealed altered basal PKA activity in cells transfected with variant-harboring *PRKAR1B* expression constructs.

**CONCLUSION:**

Our study provides strong evidence for a *PRKAR1B*-related neurodevelopmental disorder.

## INTRODUCTION

The gene *PRKAR1B* (Protein Kinase cAMP-Dependent Type I Regulatory Subunit Beta) encodes a regulatory subunit of the cyclic AMP-dependent protein kinase A protein complex (PKA), which is a nearly universal cellular component in eukaryotes.^1^ PKA is a heterotetramer of two regulatory (R) and two catalytic (C) subunits, which, upon activation of PKA by cAMP, phosphorylates serine or threonine residues of different target proteins. In humans, the genes *PRKAR1A, PRKAR1B, PRKAR2A*, and *PRKAR2B* encode the regulatory subunits RIα, RIβ, RIIα, and RIIβ, while the genes *PRKCA* and *PRKCB* give rise to a total of six principal catalytic subunit isoforms: Cα1, Cα2, and Cβ1–4. Cell type–specific expression of different subunits changes the composition and thereby intracellular localization and substrate specificity of PKA isoforms.^2^ R subunits serve as cAMP receptors and facilitate the spatial localization of PKA within the cell by binding different A-Kinase anchoring proteins (AKAPs).^3^ The subunit RIβ is primarily expressed in the brain,^4,5^ with the highest levels of expression in the cerebral cortex and hypothalamus.^6^

The first functional study of R1β involving R1β-deficient mice was completed 25 years ago, with mice deficient of the murine ortholog of R1β showing altered hippocampal long-term depression and depotentiation.^7^ Downregulation of R1β in murine hippocampal cultures was found to reduce the phosphorylation of CREB,^8^ a transcription factor implicated in long-term memory formation.^9^ Furthermore, R1β-deficient mice showed diminished nociceptive pain and inflammation in the setting of persistent tissue injury, although the reaction to acute nociceptive stimuli was unaffected.^10^

A missense variant in *PRKAR1B* has been associated with a rare hereditary neurodegenerative disorder in humans with R1β-positive inclusions in affected neurons,^11,12^ but there is also mounting evidence for a role of *PRKAR1B* in neurodevelopmental disorders (NDDs): statistical analyses of a cumulative data set of 10,927 cases derived from several NDD patient cohorts found recurrent sites (defined as “de novo missense variants of the same amino acid in two or more unrelated cases“) in *PRKAR1B*, among other potential NDD candidate genes.^13^ An analysis of expression profiles and protein–protein interaction^14^ of 253 NDD candidate genes based on the same data set positioned *PRKAR1B* among a network of genes related to c-Jun N-terminal kinase and mitogen-activated protein kinase cascades, which contained several previously identified NDD candidate genes.^15^ Another recent study found a significant enrichment of de novo missense variants in *PRKAR1B* in a large sample of 31,058 trio exomes of children with developmental disorders and their unaffected parents.^16^

*PRKAR1B* is therefore a promising candidate gene for NDDs, including autism spectrum disorder (ASD),^17^ although no clear Mendelian disease association has been established to date. We now report six unrelated individuals with variants of *PRKAR1B*, who share similar features indicative of a neurodevelopmental disorder.

## MATERIALS AND METHODS

### Exome sequencing

Trio-based exome sequencing was performed for individuals 1, 3, 4, 5, and 6. Proband-only based exome sequencing was performed for individual 2, as parental samples were not available. Individuals 1, 2, 3, 5, and 6 were enrolled through GeneDx. Individual 4 was enrolled through the University of California-Los Angeles (UCLA) Clinical Site of the Undiagnosed Diseases Network (UDN). Following informed consent, a comprehensive chart review of medical records was performed.

### DNA constructs and cell culture

The wild type (WT) (NM_001164760.2) and three variant (Glu196Lys, p.Gln167Leu, and p.Arg335Trp) *PRKAR1B* sequences were introduced into pVenus-*PRKAR1B* vector according to the method described elsewhere.^18–20^ The vector pCerulean-PRKACA was also created according to the method described elsewhere.^19^

### PKA enzymatic activity assay

HEK293 cells were transfected with the three previously described constructs (Glu196Lys, p.Gln167Leu, and p.Arg335Trp) using Lipofectamine 3000 (Invitrogen) and were harvested 24 hours post-transfection. Cells were lysed in freshly prepared lysis buffer (10 mM Tris-HCl (pH 7.5), 1 mM EDTA, and 1 mM dithiothreitol with 0.5 mM PMSF and protease inhibitor cocktail I (1:100; EMD Biosciences, La Jolla, CA). BCA assays were performed as per manufacturer’s protocol to determine the total protein concentrations of samples (Pierce). Samples were diluted to 0.5 μg/μL and 10 μL of lysate was used for each reaction. PKA enzymatic assays were performed by kemptide assay, using 25 μM kemptide (Leu-Arg-Arg-Ala-Ser-Leu-Gly), as previously described with and without cAMP (5 μM).^21^ All reactions for basal and cAMP-stimulated (total) PKA activity were carried out in duplicate. Additionally, activity values for replicate reactions that were incubated in the presence of PKI (5 nM) were subtracted from activity values to account for nonspecific kinase activity.

### Fluorescence resonance energy transfer (FRET) by acceptor photobleaching

HEK293 cells (ATCC) were seeded onto 12-well plates and left overnight to recover. The HEK293 cells were transfected with R1β-Venus and Cα-Cerulean vectors (1 μg each) and 9 hours later the cells were placed in low-serum medium (0.5% FBS) for 12 hours before stimulations. Experiments were performed on the confocal microscope Zeiss LSM 880 Airyscan as described previously (Zeiss, NY).^22^ Cα-Cerulean was imaged with the 405-nm laser, R1β-Venus with the 514-nm laser. Using a custom region of interest (ROI), R1β-Venus in one cell was bleached with the 514-nm laser at 100% transmission until the overall intensity dropped to between 80% and 50% of prebleach values. The R1β-Cα interaction was calculated by the difference in Cerulean intensity pre- versus post-bleaching. In each experiment, 10–15 bleaches on different cells were performed. Results shown are the combined results of three separate experiments.

### RNAscope in situ hybridization assay

Embryos were collected by the Human Developmental Biology Resource (https://www.hdbr.org) with ethics approval and following appropriate consent. First, 8-μm tissue sections were taken through the brain and the slides were baked for 1 hour at 60 °C before the paraffin was removed in xylene and the sections were dehydrated in two changes of 100% ethanol. Then, 1× target retrieval was performed by heating the sections for 20 minutes at 95 °C, followed by protease treatment for 15 minutes at 40 °C. An RNAscope PRKAR1β probe (ID 861041-C2) was hybridized to the tissue for 2 hours at 40 °C followed by multiple signal amplification steps. Probe hybridization was detected using Fast Red and the sections counterstained with 50% hematoxylin for 30 seconds at room temperature.

### Statistics

#### PKA enzymatic assay.

For the PKA enzymatic activity assay, data were normally distributed as determined by Shapiro–Wilk test. One-way analysis of variance (ANOVA) was performed for both the basal and total PKA activity data sets and Bonferroni multiple comparison test was used for basal activity data that produced a significant ANOVA statistic.

#### FRET experiment.

For the FRET experiment, data normality was assessed by Shapiro–Wilk test and the appropriate statistical test was used. For the FRET by acceptor photobleaching experiment, data were not normally distributed and Mann–Whitney test was used. Statistical significance was set at *P* < 0.05 and analyses were carried out using the GraphPadPrism 6 (GraphPad®) software.

#### Comparison of RNA-Seq expression levels.

For comparing RNA-Seq expression levels of different data sets from different sources^23–25^ (e.g., different experimental setup and analysis software), a normalization of the provided expression scores was necessary. We derived the distribution of expression scores over all genes and calculated the percentile of each gene of interest within this distribution. This was done individually for each data set. As a result, each scoring is limited to the range between 0% and 100%, where 0% reflects the respective gene with the lowest level of expression, and 100% the gene with the highest level of expression within the corresponding data set. The median expression level of each data set is set to 50%.

Since every source provides a single measurement of the expression levels of a certain gene, we used a bootstrapping algorithm with a sample size of 1,000 and 5 repetitions, for the derivation of the uncertainties of the resulting percentiles (error bars).

## RESULTS

We report six individuals with variants in the *PRKAR1B* gene (five males, one female; mean age 8.83 years, age range 3–16 as of June 2020). All six individuals were diagnosed with ASD by an expert physician, following DSM-V criteria. Global developmental delay (GDD) was reported in all, and congenital hypotonia was reported in three individuals. All individuals had neurologic anomalies, predominantly disorders of movement. These included dyspraxia/apraxia and clumsiness in all, tremor and dystonia in one, and involuntary movements (eye twitching) in another individual. High pain tolerance was reported by the parents of three individuals, with one individual occasionally harming himself without noticing, and another one sometimes biting himself, when frustrated, to the point of breaking the skin. Only individual 2, whose mother was also reported to have a seizure disorder, manifested seizures.

**Fig. 1:**
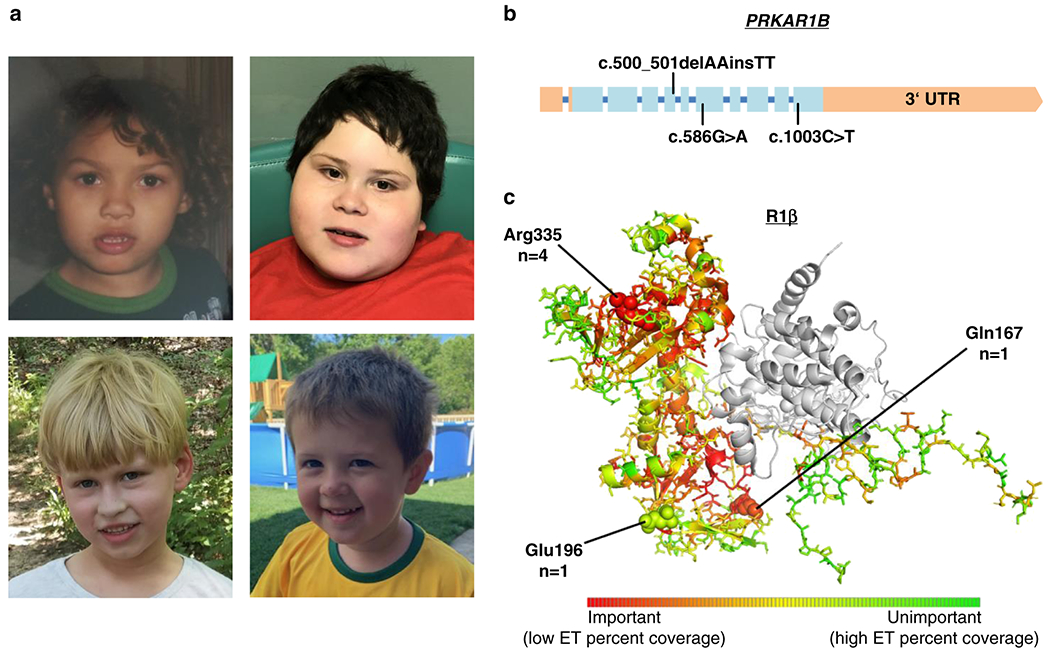
Facial phenotypes and distribution of observed *PRKAR1B* variants. (**a**) Top left: individual 1 at the age of 3 years. Top right: individual 4 the age of 7 years. Bottom left: individual 5 the age of 7 years. Bottom right: individual 6 at the age of 3 years. (**b**) Distribution of the observed variants within the *PRKAR1B* gene. Exons are shown as boxes, introns as a blue line (introns are not to scale). Light blue color indicates protein-coding sequence. (**c**) Mutated amino acid (AA) positions within the R1β protein and number of affected individuals. A color shift to red indicates a higher degree of intolerance towards AA variation throughout evolution; according to the respective position’s evolutionary trace (ET) score (see Supplemental Methods for further details).

While speech delay was reported in all individuals, speech regression has been reported in two of them: one individual lost the ability to use two-word phrases, while another one, who previously acquired an active vocabulary of ~40 words, lost most of it by age two, and became almost nonverbal from the age of five years onward. Behavioral abnormalities included autistic features like arm/hand flapping, repetitive, and sensory-seeking behavior. Attention deficit hyperactivity disorder (ADHD) was clinically diagnosed in four individuals and suspected by the parents of a fifth. Bouts of aggression were reported in three. Obesity (body mass index [BMI] > 30 kg/m^2^) was present in one individual, while another one had a BMI in the borderline obese range (BMI 29.9 kg/m^2^). Obsession with food was also reported in one of the other individuals. A compilation of phenotypic features of each individual can be found in Table 1. No consistent pattern of major malformations or physical anomalies was reported in our small cohort, although one individual had microcephaly and another one had plagiocephaly. The evaluation of facial photographs of individuals 1, 4, 5, and 6 did not reveal any consistent facial dysmorphisms other than upslanting palpebral fissures, which were seen in individuals 1, 2, 4, and 5 (Fig. 1a, Table 1).

De novo origin of the respective *PRKAR1B* variants has been confirmed in five individuals, and the parents of individual 2 were not available for testing. Four individuals carry the same variant c.1003C>T (p.Arg335Trp), while the other two carry the missense variants: c.586G>A (p.Glu196Lys) and c.500_501inv (p.Gln167Leu) (NM_001164760), respectively. A 3D model of the human R1β subunit structure^26^ is shown in Fig. 1b, highlighting the affected amino acid (AA) residues, which are situated within annotated nucleotide binding regions according to the UniProt database. All reported variants are absent from presumably healthy controls in public databases (gnomAD v2 and v3).^27^ Combined Annotation Dependent Depletion (CADD v1.6) scores^28^ calculated for the single-nucleotide variants c.1003C>T and c.586G>A were 26.4 and 24.1, respectively, ranking these variants among the most deleterious 1% of substitutions in the human genome as predicted by the CADD software. The variant c.500_501inv is situated close to the 3′ border of exon 5, and predicted functional impacts on splicing vary between the different prediction tools embedded in the Alamut Visual™ variant analysis software (v2.15), ranging from −1.6% (NNSPLICE) to −46.8% (GeneSplicer). To further estimate the functional impact, we calculated evolutionary action (EA) scores^29^ of the three variants, which predict functional impacts of AA substitutions by taking into account the phylogenetic distances of AA changes throughout the evolutionary history of the protein, as well as the compatibility of new substitutions with the typical substitutions observed between homologous sequences. The multiple sequence alignment of 106 *PRKAR1B* orthologues and paralogues used in this calculation is shown in Fig. S1. The EA scores are normalized on a scale from 0 (predicted WT protein activity) to 100 (predicted loss of protein activity), with the value representing the percentage of all possible AA substitutions in the protein that have less impact. The p.Glu196Lys, p.Gln167Leu, and p.Arg335Trp AA substitutions had EA scores of 30, 84, and 87, respectively, indicating strong functional impact for the last two, mostly due to the evolutionary pressure to preserve the Gln167 and Arg335 residues. The relatively low EA score of the p.Glu196Lys substitution is due to higher variation of this AA residue across species (67.6% Glu, 7.6% Asp, 5.7% Pro, 4.8% Ala, 4.8% Asn, 2.9% Gln, 1.9% Thr, 1.9% His, 1% Arg, and 1% Val), which however still hold a negative or neutral charge, versus the substitution of glutamic acid by positively charged lysine in the affected patient, leading to an actual alteration in charge for the respective residue.

**Fig. 2:**
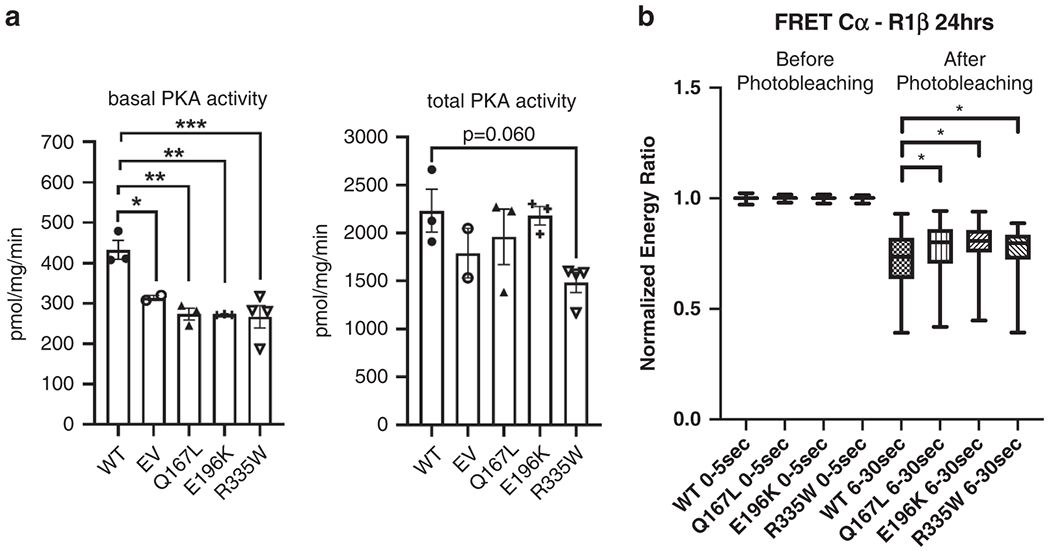
Functional consequences of the observed variants on R1β protein function. (**a**) PKA enzymatic activity assay: basal and total PKA enzymatic activity in lysates of HEK293 cells transfected with *PRKAR1B* expression constructs (wild type [WT], p.Q167L, p.E196K and p.R335W [p.Gln167Leu, p.Glu196Lys and p.Arg335Trp]). One-way analysis of variance (ANOVA) was performed for both basal and total PKA activity data sets; a Bonferroni multiple comparison test was used for basal activity data that produced a significant ANOVA statistic. (**b**) Fluorescence resonance energy transfer (FRET) in HEK293 cells transfected with R1β-Venus (WT, p.Q167L, p.E196K and p.R335W) and Cα-Cerulean vectors. A Mann–Whitney *U*-test was used to check for statistical significance as data were not normally distributed.

While in silico analyses suggested a deleterious effect of the three variants on R1β protein function, analyzing the effect of mutant *PRKAR1B* on PKA kinase in vitro could potentially validate this prediction. We performed functional studies using pVenus-*PRKAR1B* expression constructs for WT *PRKAR1B*, as well as the three variants p.Glu196Lys, p.Gln167Leu, and p.Arg335Trp. An enzymatic activity assay of lysates of HEK293 cells transfected with WT and variant-harboring constructs revealed significantly decreased basal PKA enzymatic activity in lysates of cells transfected with each of the three variant-harboring constructs (ANOVA, *p* = 0.0012). While total, or cAMP-stimulated PKA activity was not significantly different in cells transfected with WT constructs compared with those transfected with the three variant-harboring constructs (ANOVA, *p* = 0.079), the total PKA activity in lysates of cells transfected with the p.R335W construct tended to be lower (*p* = 0.060) (Fig. 2a). To explore whether the differences of basal PKA activity might be caused by impaired integration of mutant R1β into the PKA complex, fluorescence resonance energy transfer (FRET) studies were performed to detect minute positional differences of mutant and WT R1β proteins relative to the main PKA complex. In these studies, the three different variant-harboring R1β proteins appeared to bind less tightly to the main PKA catalytic subunit Cα at an approximately equal rate compared with WT R1β; The differences in the normalized energy transfer between the WT R1β-Cα interaction and the interactions of the three variant-harboring R1β proteins with Cα were small, yet significant, for all three p.Gln167Leu, p.Glu196Lys, and p.Arg335Trp variants (Fig. 2b).

**Fig. 3:**
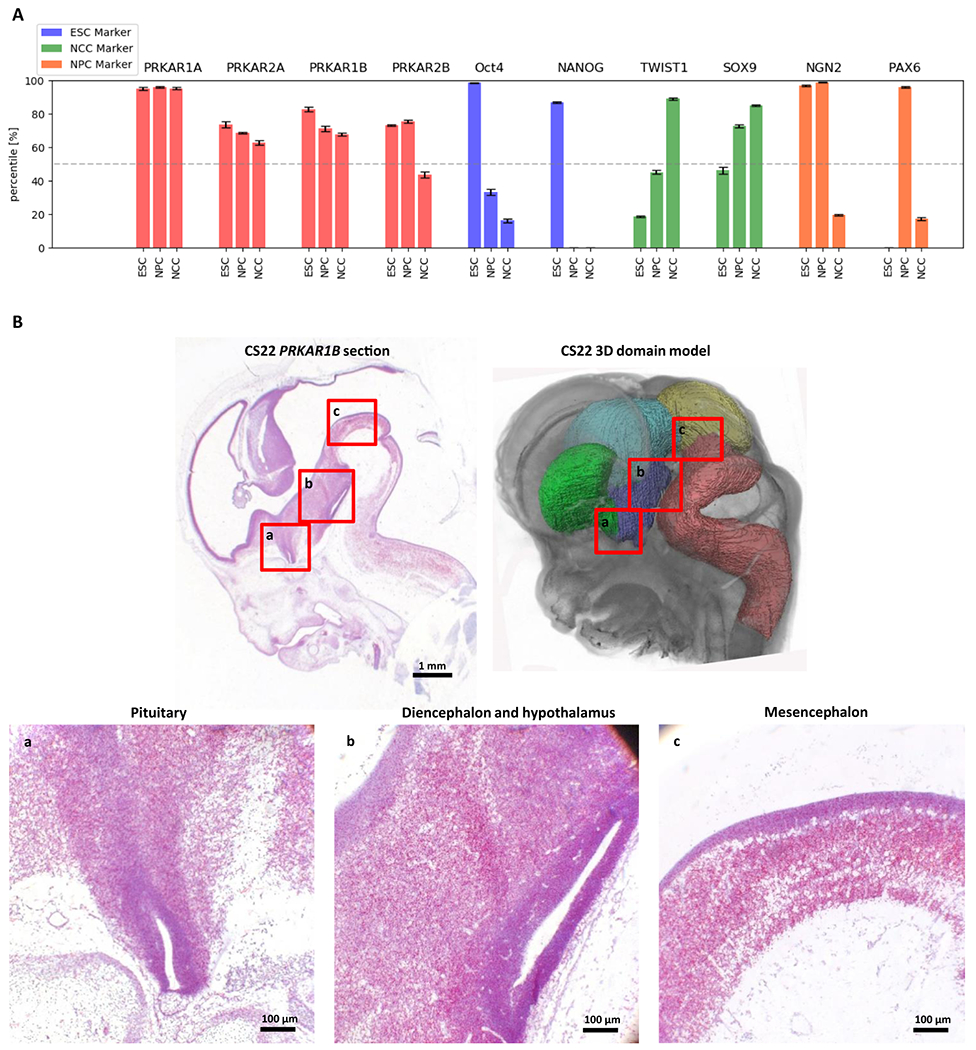
Expresssion of *PRKAR1B* during human development. (**a**) Normalized expression levels of different *PRKAR* genes and a selection of reporter genes in embryonic stem cells (ESCs), neural progenitor cells (NPCs), and neural crest cells (NCCs), based on RNA-Seq data from different sources.^23–25^ For each individual set of expression data (ESC, NPC, and NCC), 0% reflects the gene with the lowest, and 100% the gene with the highest level of expression. The median expression level of each data set is 50% (dashed line). Genes scoring higher than 50% can be considered to be more highly expressed than the majority of genes in their respective data set. (**b**) Upper row: sagittal section of a human embryo at Carnegie stage 22 and corresponding 3D model of the embryonic brain (yellow: mesencephalon; green: subpallium; light blue: diencephalon; purple: hypothalamus; pink: rhombencephalon). A RNAscope PRKAR1β probe has been used to hybridize *PRKAR1B* messenger RNA (mRNA) (red). The section has been counterstained with hematoxylin (blue). A corresponding positive and negative control is shown in Fig. S2. Lower row: magnified sections show PRKAR1B expression in the pituitary, diencephalon, mesencephalon, and hypothalamus.

As a potential NDD disease gene, *PRKAR1B* would be expected to be expressed in the brain during embryonal development. *PRKAR1B* expression is seen in different embryonic cell types including neural progenitor cells (NPCs) and neural crest cells (NCCs) (Fig. 3a).^23–25^ Additionally, an in situ hybridization assay to detect *PRKAR1B* messenger RNA (mRNA) (RNAscope) revealed high *PRKAR1B* expression in the pituitary, diencephalon, mesencephalon, and hypothalamus in human embryos at Carnegie stage 22 (Fig. 3b).

## DISCUSSION

This study presents a systematic characterization of a NDD associated with missense variants in *PRKAR1B*. In addition to the observed enrichment of de novo missense variants of this gene in two large, independent cohorts of individuals with NDDs,^15,16^ the recurrent finding of the de novo variant c.1003C>T in phenotypically similar individuals strongly suggests that this variant is causative for the observed phenotype (see Supplemental Note 1), and indicates a potential mutational hotspot in the Arg335 residue. The observation of altered basal PKA activity in HEK293 cells transfected with variant-harboring constructs supports the hypothesis of a deleterious effect of the reported *PRKAR1B* variants on PKA function. It is important to note, however, that a decreased basal PKA enzymatic activity has been associated with a tighter binding of R1β to the Cα subunit in past studies.^20^ It is therefore possible that the small differences between the WT and variant-harboring R1β subunits identified by FRET do not actually cause a weaker R1β-Cα interaction, but may rather point toward another, yet unknown, molecular disease mechanism.

While expression of *PRKAR1B* in the adult brain had been previously shown,^5^ the high expression of the gene in the embryonic brain at CS22 points toward an early role of *PRKAR1B* in the development of the brain, which would be consistent with the concept of a *PRKAR1B*-associated neurodevelopmental disorder.

The clinical features of our cohort seem to approximate some aspects of the phenotype of R1β-deficient mice, such as increased pain tolerance,^10^ which was reported in three patients carrying the c.1003C>T variant. Furthermore, defects in hippocampal homosynaptic long-term depotentiation and low frequency stimulus-induced synaptic depression reported in mice,^7^ if also present in humans, may influence synaptic plasticity and thereby potentially impair learning and other cognitive functions. On a cellular level, cognitive abnormalities may be caused by diminished PKA-mediated phosphorylation of CREB in neurons. As the cAMP/PKA/CREB cascade is instrumental for the transcription of memory-associated genes and long-term memory formation,^9,30^ further studies might demonstrate functional impacts of *PRKAR1B* variants by measuring CREBP phosphorylation and expression of target genes in human IPSC-derived neurons from affected individuals and healthy controls.

We propose a *PRKAR1B*-associated NDD with GDD, ASD, neurologic anomalies, and cognitive impairment (no formal IQ scores have yet been obtained from the reported individuals) as principal features. Future research should focus on better understanding the functional consequences of *PRKAR1B* variants, as well as the recruitment and clinical characterization of more individuals carrying de novo *PRKAR1B* variants.

## Supplementary Material

1704050_Supp_Info

## Figures and Tables

**Table 1.** Genotypes and phenotypic features of individuals 1–6.

Individual	1	2	3	4	5	6
Age/sex/BMI	13 years/M/unknown	16 years/F/29.9	7 years/M/unknown	7 years/M/32.5	7 years/M/17.3	3 years/M/17.9
Variant (NM_001164760)	c.586G>A, p.Glu196Lys	c.500_501inv, p.Gln167Leu	c.1003C>T, p.Arg335Trp	c.1003C>T, p.Arg335Trp	c.1003C>T, p.Arg335Trp	c.1003C>T, p.Arg335Trp
De novo?	Yes	Unknown	Yes	Yes	Yes	Yes
Global developmental delay	Yes	Yes	Yes	Yes	Yes	Yes
Diagnosed ASD	Yes	Yes	Yes	Yes	Yes	Yes
ADHD	Yes	Unknown	Yes	Suspected by parents, no formal diagnosis	Yes	Yes
Dyspraxia /apraxia	Yes	Yes	Yes	Yes	Yes	Yes
Congenital hypotonia	Unknown	Unknown	Unknown	Yes	Yes	Yes
Speech	Speech delay	Speech delay	Speech delay	Speech regression, nonverbal	Speech delay, 1–3 word sentences	Speech delay and regression, single words
Other behavioral anomalies	Aggression	Immature for age	No	Aggression, hand flapping	Arm flapping, aggression when frustrated	Odd/repetitive behaviors, arm flapping, happy demeanor, sensory-seeking
Pain tolerance	Normal	Unknown	Unknown	High	Very high	High
Other neurologic anomalies	Tremor, Hemidystonia	Seizures	No	No	No	Eye twitching
Physical anomalies	Upslanting palpebral fissures	Upslanting palpebral fissures, other dysmorphic features^a^	No	Obesity, astigmatism, esotropia, upslanting palpebral fissures, other dysmorphic features^b^	Microcephaly, upslanting palpebral fissures	Torticollis, plagiocephaly, submucosal cleft palate dysmorphic features^c^
Skill regression	No	Unknown	No	No	No	Potential skill regression and plateauing of progress
Other features	Unknown	Asthma, fatigue	Sleep problems, nocturnal enuresis	Obstructive sleep apnea, recurrent otitis media	Sleep disturbance, severe eczema^d^	Restlessness, obstructive sleep apnea

*ADHD* attention deficit hyperactivity disorder, *ASD* autism spectrum disorder, *BMI* body mass index.

a Round face, broad nasal tip, thin upper lip, and short palpebral fissures.

b Hypotelorism, bitemporal narrowing, epicanthal folds, flat nasal bridge, downturned mouth, tapered fingers, doughy hands, and brachydactyly (Fig. 1a).

c Epicanthal folds, slightly posteriorly rotated ears (Fig. 1a).

d Individual 5 is also homozygous for a pathogenic variant in *FLG* (c.2282_2285delCAGT; p.Ser761Cysfs*36), which explains his severe eczema and dry skin.

## UNDIAGNOSED DISEASES NETWORK

Maria T. Acosta^17^, Margaret Adam^18^, David R. Adams^17^, Pankaj B. Agrawal^19^, Mercedes E. Alejandro^20^, Justin Alvey^21^, Laura Amendola^18^, Ashley Andrews^21^, Euan A. Ashley^22^, Mahshid S. Azamian^20^, Carlos A. Bacino^20^, Guney Bademci^23^, Eva Baker^17^, Ashok Balasubramanyam^20^, Dustin Baldridge^24,25^, Jim Bale^21^, Michael Bamshad^18^, Deborah Barbouth^23^, Pinar Bayrak-Toydemir^21^, Anita Beck^18^, Alan H. Beggs^19^, Edward Behrens^26^, Gill Bejerano^22^, Jimmy Bennett^18^, Beverly Berg-Rood^18^, Jonathan A. Bernstein^22^, Gerard T. Berry^19^, Anna Bican^27^, Stephanie Bivona^23^, Elizabeth Blue^18^, John Bohnsack^21^, Carsten Bonnenmann^17^, Devon Bonner^22^, Lorenzo Botto^21^, Brenna Boyd^18^, Lauren C. Briere^19^, Elly Brokamp^27^, Gabrielle Brown^28^, Elizabeth A. Burke^17^, Lindsay C. Burrage^20^, Manish J. Butte^28^, Peter Byers^18^, William E. Byrd^29^, John Carey^21^, Olveen Carrasquillo^23^, Ta Chen Peter Chang^23^, Sirisak Chanprasert^18^, Hsiao-Tuan Chao^20^, Gary D. Clark^20^, Terra R. Coakley^22^, Laurel A. Cobban^19^, Joy D. Cogan^27^, Matthew Coggins^19^, F. Sessions Cole^24^, Heather A. Colley^17^, Cynthia M. Cooper^19^, Heidi Cope^30^, William J. Craigen^20^, Andrew B. Crouse^29^, Michael Cunningham^18^, Precilla D’Souza^17^, Hongzheng Dai^20^, Surendra Dasari^31^, Joie Davis^17^, Jyoti G. Daya^17^, Matthew Deardorff^26^, Esteban C. Dell’Angelica^28^, Shweta U. Dhar^20^, Katrina Dipple^18^, Daniel Doherty^18^, Naghmeh Dorrani^28^, Argenia L. Doss^17^, Emilie D. Douine^28^, David D. Draper^17^, Laura Duncan^27^, Dawn Earl^18^, David J. Eckstein^17^, Lisa T. Emrick^20^, Christine M. Eng^32^, Cecilia Esteves^33^, Marni Falk^26^, Liliana Fernandez^22^, Carlos Ferreira^17^, Elizabeth L. Fieg^19^, Laurie C. Findley^17^, Paul G. Fisher^22^, Brent L. Fogel^28^, Irman Forghani^23^, Laure Fresard^22^, William A. Gahl^17^, Ian Glass^18^, Bernadette Gochuico^17^, Rena A. Godfrey^17^, Katie Golden-Grant^18^, Alica M. Goldman^20^, Madison P. Goldrich^17^, David B. Goldstein^34^, Alana Grajewski^23^, Catherine A. Groden^17^, Irma Gutierrez^28^, Sihoun Hahn^18^, Rizwan Hamid^27^, Neil A. Hanchard^20^, Kelly Hassey^26^, Nichole Hayes^24^, Frances High^19^, Anne Hing^18^, Fuki M. Hisama^18^, Ingrid A. Holm^19^, Jason Hom^22^, Martha Horike-Pyne^18^, Alden Huang^28^, Yong Huang^22^, Laryssa Huryn^17^, Rosario Isasi^23^, Fariha Jamal^20^, Gail P. Jarvik^18^, Jeffrey Jarvik^18^, Suman Jayadev^18^, Lefkothea Karaviti^20^, Jennifer Kennedy^27^, Dana Kiley^24^, Isaac S. Kohane^33^, Jennefer N. Kohler^22^, Susan Korrick^19^, Mary Kozuira^27^, Deborah Krakow^28^, Donna M. Krasnewich^17^, Elijah Kravets^22^, Joel B. Krier^19^, Grace L. LaMoure^17^, Seema R. Lalani^20^, Byron Lam^23^, Christina Lam^18^, Brendan C. Lanpher^31^, Ian R. Lanza^31^, Lea Latham^17^, Kimberly LeBlanc^33^, Brendan H. Lee^20^, Hane Lee^28^, Roy Levitt^23^, Richard A. Lewis^20^, Sharyn A. Lincoln^19^, Pengfei Liu^32^, Xue Zhong Liu^23^, Nicola Longo^21^, Sandra K. Loo^28^, Joseph Loscalzo^19^, Richard L. Maas^19^, John MacDowall^17^, Calum A. MacRae^19^, Ellen F. Macnamara^17^, Valerie V. Maduro^17^, Marta M. Majcherska^22^, Bryan C. Mak^28^, May Christine V. Malicdan^17^, Laura A. Mamounas^17^, Teri A. Manolio^17^, Rong Mao^21^, Kenneth Maravilla^18^, Thomas C. Markello^17^, Ronit Marom^20^, Gabor Marth^21^, Beth A. Martin^22^, Martin G. Martin^28^, Julian A. Martinez-Agosto^28^, Shruti Marwaha^22^, Jacob McCauley^23^, Allyn McConkie-Rosell^30^, Colleen E. McCormack^22^, Alexa T. McCray^33^, Elisabeth McGee^28^, Heather Mefford^18^, J. Lawrence Merritt^18^, Matthew Might^29^, Ghayda Mirzaa^18^, Eva Morava^31^, Paolo M. Moretti^20^, Paolo Moretti^21^, Deborah Mosbrook-Davis^17^, John J. Mulvihill^17^, David R. Murdock^20^, Anna Nagy^33^, Mariko Nakano-Okuno^29^, Avi Nath^17^, Stanley F. Nelson^28^, John H. Newman^27^, Sarah K. Nicholas^20^, Deborah Nickerson^18^, Shirley Nieves-Rodriguez^28^, Donna Novacic^17^, Devin Oglesbee^31^, James P. Orengo^20^, Laura Pace^21^, Stephen Pak^25^, J. Carl Pallais^19^, Christina G. S. Palmer^28^, Jeanette C. Papp^28^, Neil H. Parker^28^, John A. Phillips III^27^, Jennifer E. Posey^20^, Lorraine Potocki^20^, Bradley Power^17^, Barbara N. Pusey^17^, Aaron Quinlan^21^, Archana N. Raja^22^, Deepak A. Rao^19^, Wendy Raskind^18^, Genecee Renteria^28^, Chloe M. Reuter^22^, Lynette Rives^27^, Amy K. Robertson^27^, Lance H. Rodan^19^, Jill A. Rosenfeld^20^, Natalie Rosenwasser^18^, Francis Rossignol^17^, Maura Ruzhnikov^22^, Ralph Sacco^23^, Jacinda B. Sampson^22^, Susan L. Samson^20^, Mario Saporta^23^, Judy Schaechter^23^, Timothy Schedl^25^, Kelly Schoch^30^, C. Ron Scott^17^, Daryl A. Scott^20^, Vandana Shashi^30^, Jimann Shin^25^, Rebecca H. Signer^28^, Edwin K. Silverman^19^, Janet S. Sinsheimer^28^, Kathy Sisco^24^, Edward C. Smith^30^, Kevin S. Smith^22^, Emily Solem^27^, Lilianna Solnica-Krezel^25^, Ben Solomon^17^, Rebecca C. Spillmann^30^, Joan M. Stoler^19^, Jennifer A. Sullivan^30^, Kathleen Sullivan^26^, Angela Sun^18^, Shirley Sutton^22^, David A. Sweetser^19^, Virginia Sybert^18^, Holly K. Tabor^22^, Amelia L. M. Tan^33^, Queenie K.-G. Tan^30^, Mustafa Tekin^23^, Fred Telischi^23^, Willa Thorson^23^, Audrey Thurm^17^, Cynthia J. Tifft^17^, Camilo Toro^17^, Alyssa A. Tran^20^, Brianna M. Tucker^22^, Tiina K. Urv^17^, Adeline Vanderver^26^, Matt Velinder^21^, Dave Viskochil^21^, Tiphanie P. Vogel^20^, Colleen E. Wahl^17^, Melissa Walker^19^, Stephanie Wallace^18^, Nicole M. Walley^30^, Chris A. Walsh^19^, Jennifer Wambach^24^, Jijun Wan^28^, Lee-kai Wang^28^, Michael F. Wangler^35^, Patricia A. Ward^32^, Daniel Wegner^24^, Mark Wener^18^, Tara Wenger^18^, Katherine Wesseling Perry^28^, Monte Westerfield^36^, Matthew T. Wheeler^22^, Jordan Whitlock^29^, Lynne A. Wolfe^17^, Jeremy D. Woods^28^, Shinya Yamamoto^35^, John Yang^17^, Muhammad Yousef^17^, Diane B. Zastrow^22^, Wadih Zein^17^, Chunli Zhao^22^ and Stephan Zuchner^23^

^17^National Institutes of Health, Undiagnosed Diseases Program Clinical Site, Bethesda, MD, USA. ^18^University of Washington and Seattle Children’s Hospital Clinical Site, Seattle, WA, USA. ^19^Harvard-affiliated Boston Children’s Hospital, Massachusetts General Hospital, Brigham and Women’s Hospital, and Brigham Genomics Medicine Clinical Site, Boston, MA, USA. ^20^Baylor College of Medicine, Clinical Site, Houston, TX, USA. ^21^University of Utah Clinical Site, Salt Lake City, UT, USA. ^22^Stanford University Clinical Site, Stanford, CA, USA. ^23^University of Miami Clinical Site, Miami, FL, USA. ^24^Washington University of Saint Louis, Clinical Site, Saint Louis, MO, USA. ^25^Washington University of Saint Louis, Model Organism Screening Center, Saint Louis, MO, USA. ^26^Children’s Hospital of Philadelphia or University of Pennsylvania Clinical Site, Philadelphia, PA, USA. ^27^Vanderbilt University Clinical Site, Nashville, TN, USA. ^28^University of California, Los Angeles, Clinical Site, Los Angeles, CA, USA. ^29^University of Alabama Coordinating Center, Birmingham, AL, USA. ^30^Duke University Clinical Site, Durham, NC, USA. ^31^Mayo Clinic Metabolomics Core, Rochester, MN, USA. ^32^Baylor Genetics Sequencing Core, Houston, TX, USA. ^33^Harvard Medical School Coordinating Center, Boston, MA, USA. ^34^Columbia University Clinical Site, New York City, NY, USA. ^35^Baylor College of Medicine, Model Organism Screening Center, Houston, TX, USA. ^36^University of Oregon, Model Organism Screening Center, Eugene, OR, USA.
