# Supplementary material for: Variants in PRKAR1B cause a neurodevelopmental disorder with autism spectrum disorder, apraxia, and insensitivity to pain
Source: Genet Med. Author manuscript; Available in PMC 2021 Aug 14. (PMC8354857; doi:10.1038/s41436-021-01152-7)
Supplement: 1704050_Supp_Info [file NIHMS1704050-supplement-1704050_Supp_Info.pdf]

Supplement

Figure S1 - Phylogenetic tree of the homologous *PRKAR1B* sequences Page 2

Figure S2 – RNAscope imaging: Positive and negative control sections Page 3

Supplemental Note 1: Comment on *de novo* SNVs Page 4

Supplemental Methods Page 5

Supplemental References Page 6

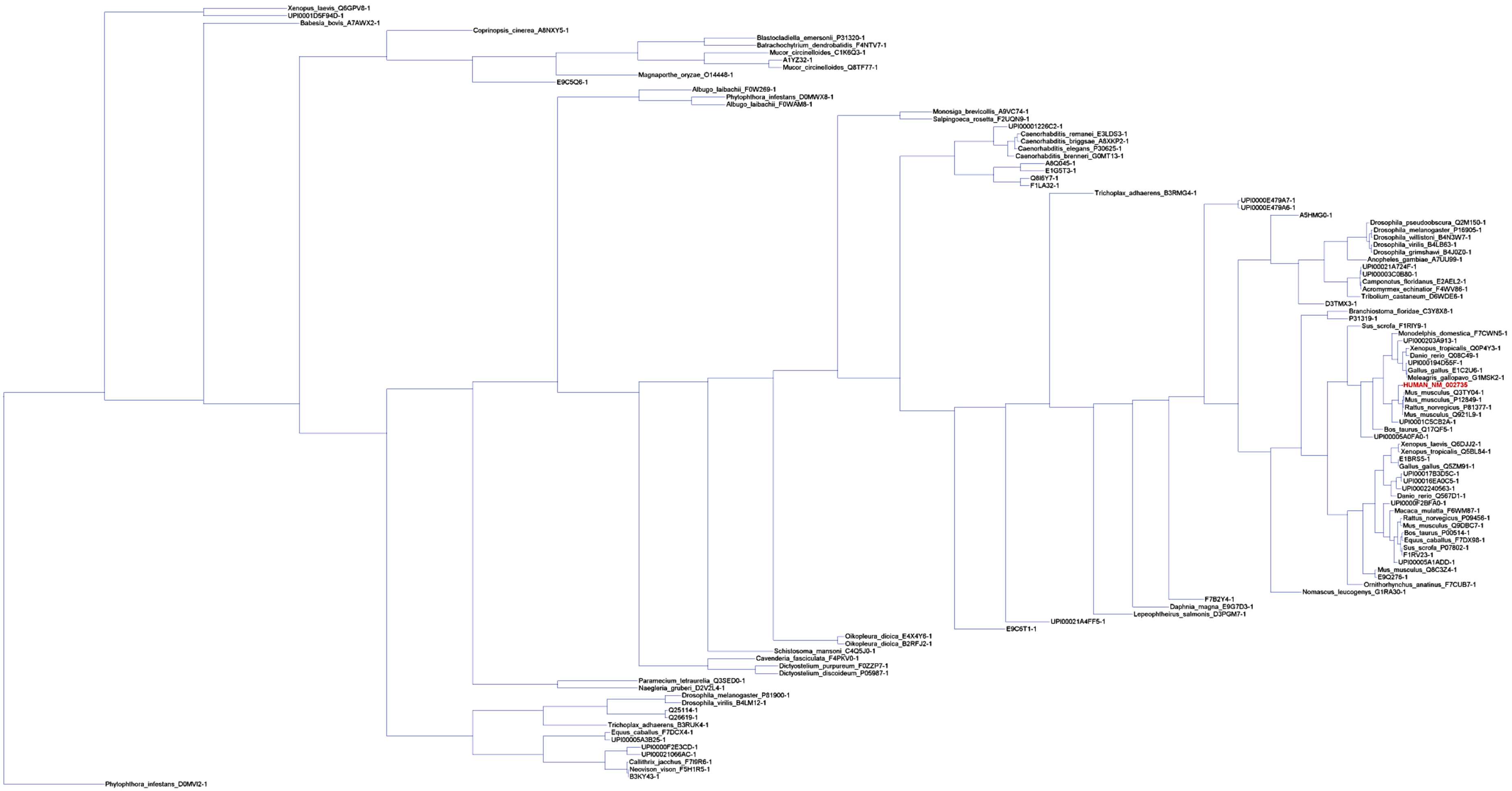
Xenopus_laevis_Q6GPV8-1
UPI0001D5F94D-1
Babesia_bovis_A7AWX2-1
Coprinopsis_cinerea_A8NXY5-1
Blastocladiella_emersonii_P31320-1
Batrachochytrium_dendrobatidis_F4NTV7-1
Mucor_circinelloides_C1K6Q3-1
A1YZ32-1
Mucor_circinelloides_Q8TF77-1
Magnaporthe_oryzae_O14448-1
E9C5Q6-1
Albugo_laibachii_F0W269-1
Phytophthora_infestans_D0MWX8-1
Albugo_laibachii_F0WAM8-1
Monosiga_brevicollis_A9VC74-1
Salpingoeca_rosetta_F2UQN9-1
UPI00001226C2-1
Caenorhabditis_remanei_E3LDS3-1
Caenorhabditis_briggsae_A8XKP2-1
Caenorhabditis_elegans_P30625-1
Caenorhabditis_brenneri_G0MT13-1
A8Q045-1
E1G5T3-1
Q8I6Y7-1
F1LA32-1
Trichoplax_adhaerens_B3RMG4-1
UPI0000E479A7-1
UPI0000E479A6-1
A5HMG0-1
Drosophila_pseudoobscura_Q2M150-1
Drosophila_melanogaster_P16905-1
Drosophila_willistoni_B4N3W7-1
Drosophila_virilis_B4LB63-1
Drosophila_grimshawi_B4J0Z0-1
Anopheles_gambiae_A7UU99-1
UPI00021A724F-1
UPI00003C0B80-1
Camponotus_floridanus_E2AEL2-1
Acromyrmex_echinatior_F4WV86-1
Tribolium_castaneum_D6WDE6-1
D3TMX3-1
Branchiostoma_floridae_C3Y8X8-1
P31319-1
Sus_scrofa_F1RIY9-1
Monodelphis_domestica_F7CWN5-1
UPI000203A913-1
Xenopus_tropicalis_Q0P4Y3-1
Danio_rerio_Q08C49-1
UPI000194D55F-1
Gallus_gallus_E1C2U6-1
Meleagris_gallopavo_G1MSK2-1
HUMAN_NM_002735
Mus_musculus_Q3TY04-1
Mus_musculus_P12849-1
Rattus_norvegicus_P81377-1
Mus_musculus_Q921L9-1
UPI0001C5CB2A-1
Bos_taurus_Q17QF5-1
UPI00005A0FA0-1
Xenopus_laevis_Q6DJJ2-1
Xenopus_tropicalis_Q5BL84-1
E1BRS5-1
Gallus_gallus_Q5ZM91-1
UPI00017B3D5C-1
UPI00016EA0C5-1
UPI0002240563-1
Danio_rerio_Q567D1-1
UPI0000F2BFA0-1
Macaca_mulatta_F6WM87-1
Rattus_norvegicus_P09456-1
Mus_musculus_Q9DBC7-1
Bos_taurus_P00514-1
Equus_caballus_F7DX98-1
Sus_scrofa_P07802-1
F1RV23-1
UPI00005A1ADD-1
Mus_musculus_Q8C3Z4-1
E9Q276-1
Ornithorhynchus_anatinus_F7CUB7-1
Nomascus_leucogenys_G1RA30-1
F7B2Y4-1
Daphnia_magna_E9G7D3-1
Lepeophtheirus_salmonis_D3PGM7-1
UPI00021A4FF5-1
E9C6T1-1
Oikopleura_dioica_E4X4Y6-1
Oikopleura_dioica_B2RFJ2-1
Schistosoma_mansoni_C4Q5J0-1
Cavenderia_fasciculata_F4PKV0-1
Dictyostelium_purpureum_F0ZZP7-1
Dictyostelium_discoideum_P05987-1
Paramecium_tetraurelia_Q3SED0-1
Naegleria_gruberi_D2V2L4-1
Drosophila_melanogaster_P81900-1
Drosophila_virilis_B4LM12-1
Q25114-1
Q26619-1
Trichoplax_adhaerens_B3RUK4-1
Equus_caballus_F7DCX4-1
UPI00005A3B25-1
UPI0000F2E3CD-1
UPI00021066AC-1
Callithrix_jacchus_F7I9R6-1
Neovison_vison_F5H1R5-1
B3KY43-1
Phytophthora_infestans_D0MVI2-1

Figure S1

The phylogenetic tree of the homologous *PRKAR1B* sequences obtained from the UniRef100 database. The sequences were selected to represent different depths of the evolutionary history of this gene and they include both orthologous and paralogous sequences. The human sequence is highlighted with red. This alignment was automatically generated as follows: A protein BLAST search[1] of the query sequence on the UniRef100 database[2] was performed. These 106 sequences were chosen to represent different evolution depths and they were aligned with the program MUSCLE[3]. The phylogenetic tree was created by PILEUP[4] which is embodied to the Evolutionary Trace algorithm[5]. The phylogenetic tree was visualized using Archaeopteryx (version 0.9901).

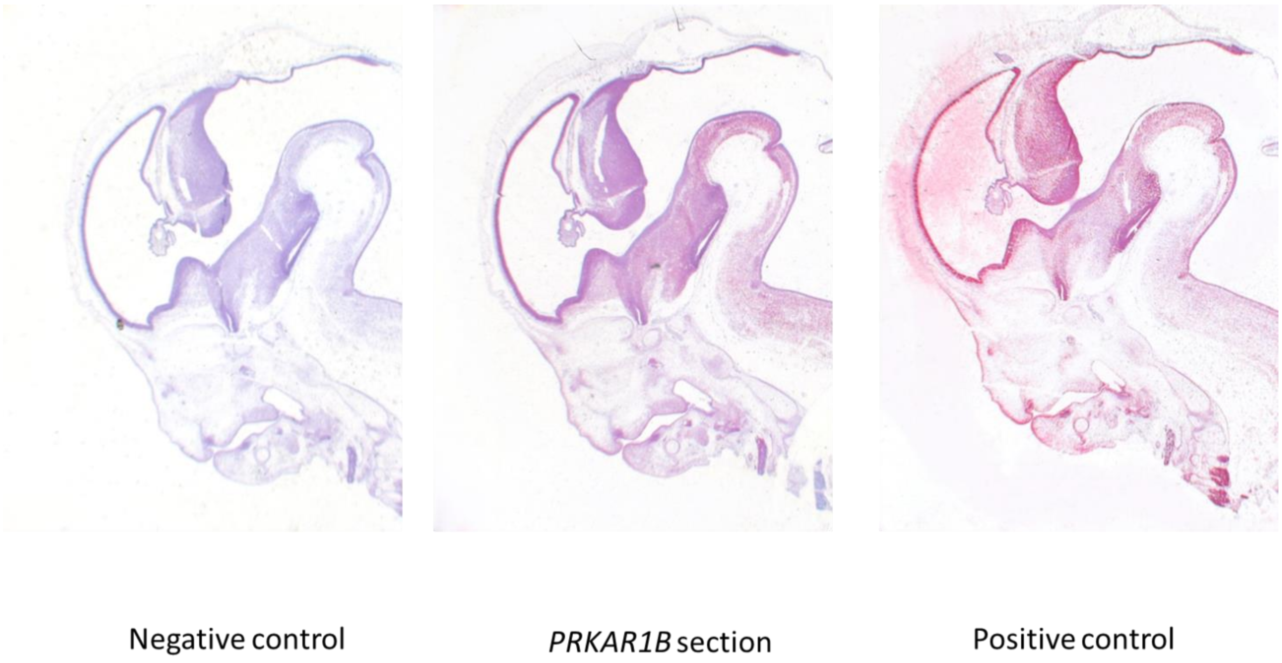

Figure S2

Sagittal sections of a human embryo at CS22. Left and right: Corresponding negative and positive control sections to the section shown in Figure 3B (middle).

Supplemental Note 1: Comment on *de novo* SNVs

The average *de novo* mutation rate in the human genome lies between 1.0×10^{-8} and 2.5×10^{-8} per base pair per generation[6-10]. However, the mutation rate varies strongly depending on sequence context[6; 10]. The mutation rate for C>T transitions at CpG sites is significantly higher than the average, estimated at 1.12×10^{-7} per base pair per generation[6]. As the *PRKAR1B* c.1003C>T variant is a C>T transition in a CpG context, we investigated the likelihood of observing this identical *de novo* transition in multiple unrelated patients. The likelihood for any single phenotypically similar individual to carry the identical *de novo* exonic CpG-context transition by chance alone would similarly be 1.12×10^{-7}, or about 1 in 8.9 million. We used the bedtools v2.26.0 intersectBed tool in conjunction with the Agilent SureSelect All Exon v7 target region BED to identify 1,149,279 exonic CpG sites in the human hg19 reference genome. This translates to 2,298,558 nucleotides in a CpG context with the abovementioned higher mutation rate. We would therefore expect on the order of 0.26 *de novo* CpG-context transitions in a single individual's exome.

The *de novo* c.1003C>T variant in *PRKAR1B* was observed in three patients in the GeneDx diagnostic cohort of patients with developmental disorders. This cohort contains 31,058 trio-exome datasets and in it, we would thus expect around 7,995 *de novo* CpG-context transitions. By chance alone, the likelihood of observing the *PRKAR1B* c.1003C>T transition in two or more unrelated, phenotypically similar patients from this cohort after the initial observation in the first patient would be 6.04×10^{-6}, or about 1 in 166,000 (binomial test).

Supplemental Methods

Visualization of the Protein Structure

We obtained the crystallographic 3D structure of the R1β subunit from the Protein Data Bank (ID of 4DIN; chain B)[11]. This structure represents the human protein of PRKAR1B bound to the mice protein of Pkaca (homolog of human PRKACA). Pymol 2.3.4 was used to visualize the structure (PyMOL Molecular Graphics System, Version 2.0 Schrödinger, LLC.). The structure was colored according to the Evolutionary Trace scores[5; 12], using PyETV[13].

Evolutionary Action (EA) Scores

The EA scores were calculated for the protein sequence NP_002726[14]. The input consisted from 200 homologous sequences to the human R1β subunit, including sequences from species as distant as Oomycetes. Briefly, the EA scores estimate the effect of the variants in protein fitness and they are given in a scale from 0 (wild type) to 100 (loss of function). Previous work has shown that the EA scores correlate with the percent loss of protein function in experimental assays (or the probability to be found deleterious in binary assays) and with clinical associations to disease[15; 16]. EA scores are available at **http://eaction.lichtargelab.org/**.

Supplemental references

1. Altschul, S.F., Madden, T.L., Schaffer, A.A., Zhang, J., Zhang, Z., Miller, W., and Lipman, D.J. (1997). Gapped BLAST and PSI-BLAST: a new generation of protein database search programs. Nucleic Acids Res 25, 3389-3402.
2. Suzek, B.E., Wang, Y., Huang, H., McGarvey, P.B., Wu, C.H., and UniProt, C. (2015). UniRef clusters: a comprehensive and scalable alternative for improving sequence similarity searches. Bioinformatics 31, 926-932.
3. Edgar, R.C. (2004). MUSCLE: multiple sequence alignment with high accuracy and high throughput. Nucleic Acids Res 32, 1792-1797.
4. Feng, D.F., and Doolittle, R.F. (1987). Progressive sequence alignment as a prerequisite to correct phylogenetic trees. J Mol Evol 25, 351-360.
5. Lichtarge, O., Bourne, H.R., and Cohen, F.E. (1996). An evolutionary trace method defines binding surfaces common to protein families. J Mol Biol 257, 342-358.
6. Kong, A., Frigge, M.L., Masson, G., Besenbacher, S., Sulem, P., Magnusson, G., Gudjonsson, S.A., Sigurdsson, A., Jonasdottir, A., Jonasdottir, A., et al. (2012). Rate of de novo mutations and the importance of father's age to disease risk. Nature 488, 471-475.
7. Conrad, D.F., Keebler, J.E., DePristo, M.A., Lindsay, S.J., Zhang, Y., Casals, F., Idaghdour, Y., Hartl, C.L., Torroja, C., Garimella, K.V., et al. (2011). Variation in genome-wide mutation rates within and between human families. Nat Genet 43, 712-714.
8. Sun, J.X., Helgason, A., Masson, G., Ebenesersdottir, S.S., Li, H., Mallick, S., Gnerre, S., Patterson, N., Kong, A., Reich, D., et al. (2012). A direct characterization of human mutation based on microsatellites. Nat Genet 44, 1161-1165.
9. Michaelson, J.J., Shi, Y., Gujral, M., Zheng, H., Malhotra, D., Jin, X., Jian, M., Liu, G., Greer, D., Bhandari, A., et al. (2012). Whole-genome sequencing in autism identifies hot spots for de novo germline mutation. Cell 151, 1431-1442.
10. Besenbacher, S., Sulem, P., Helgason, A., Helgason, H., Kristjansson, H., Jonasdottir, A., Jonasdottir, A., Magnusson, O.T., Thorsteinsdottir, U., Masson, G., et al. (2016). Multi-nucleotide de novo Mutations in Humans. PLoS Genet 12, e1006315.
11. Ilouz, R., Bubis, J., Wu, J., Yim, Y.Y., Deal, M.S., Kornev, A.P., Ma, Y., Blumenthal, D.K., and Taylor, S.S. (2012). Localization and quaternary structure of the PKA RIbeta holoenzyme. Proc Natl Acad Sci U S A 109, 12443-12448.
12. Mihalek, I., Res, I., and Lichtarge, O. (2004). A family of evolution-entropy hybrid methods for ranking protein residues by importance. J Mol Biol 336, 1265-1282.
13. Lua, R.C., and Lichtarge, O. (2010). PyETV: a PyMOL evolutionary trace viewer to analyze functional site predictions in protein complexes. Bioinformatics 26, 2981-2982.
14. Katsonis, P., and Lichtarge, O. (2014). A formal perturbation equation between genotype and phenotype determines the Evolutionary Action of protein-coding variations on fitness. Genome Res 24, 2050-2058.
15. Katsonis, P., and Lichtarge, O. (2017). Objective assessment of the evolutionary action equation for the fitness effect of missense mutations across CAGI-blinded contests. Hum Mutat 38, 1072-1084.
16. Katsonis, P., and Lichtarge, O. (2019). CAGI5: Objective performance assessments of predictions based on the Evolutionary Action equation. Hum Mutat 40, 1436-1454.
